# Biomarkers and arterial stiffness. A sub-analysis of the “Exercise training in heart failure with reduced ejection fraction and permanent atrial fibrillation: A randomized clinical trial”

**DOI:** 10.1016/j.clinsp.2026.101142

**Published:** 2026-09-10

**Authors:** Guilherme Veiga Guimarães, Veridiana Moraes D’ Avila, Paulo Roberto Chizzola, Cristian Alvarez, Edimar Alcides Bocchi

**Affiliations:** a Instituto do Coração, Hospital das Clínicas da Faculdade de Medicina da Universidade de São Paulo, São Paulo, SP, Brazil; b Exercise and Rehabilitation Sciences Institute, School of Physical Therapy, Faculty of Rehabilitation Sciences, Universidad Andres Bello, Santiago, Chile

**Keywords:** Atrial fibrillation, Heart failure, Exercise training, Arterial stiffness, Biomarkers

## Abstract

**Objective:**

Regular exercise improves outcomes in patients with Heart Failure (HF) and Atrial Fibrillation (AF), but its effects on inflammatory pathways and arterial stiffness in HF with Reduced Ejection Fraction (HFrEF) and permanent AF remain uncertain. This study examined the effects of a 12-week aerobic exercise program on biomarkers and arterial stiffness.

**Design and methods:**

This secondary analysis was derived from a parallel group randomized trial. We recruited 32 patients with HFrEF and permanent AF, randomizing 1:1 to an exercise group (n = 16) or a control group (n = 16). Six participants discontinued the protocol (two dropouts and one death per group), resulting in a final analyzed cohort of 26 patients who completed the study (13-per-group). The exercise group performed supervised aerobic training three times weekly for 12-weeks, while controls received usual care. Levels of BNP, ANP, IL-6, TNF-α, angiotensin II, and arterial stiffness by pulse wave velocity were measured at baseline and post-intervention.

**Results:**

Baseline characteristics were comparable. After 12-weeks, the exercise group showed significant reductions in BNP, ANP, IL-6, TNF-α, and angiotensin II, whereas the control group exhibited significant increases in ANP, IL-6, TNF-α, and angiotensin II. Arterial stiffness remained unchanged in the exercise group but increased in controls; however, the between-group difference was not statistically significant.

**Conclusion:**

A 12-week aerobic exercise program favorably modulated inflammatory and neurohormonal biomarkers in patients with HFrEF and permanent AF; however, its effect on arterial stiffness was less clear. Although arterial stiffness progressed in the control group and remained stable in the exercise group, the between-group difference in this change was not statistically significant. As these findings derive from a secondary analysis of a randomized trial, they should be interpreted with caution.

## Highlights

• Exercise training improves biomarkers in HFrEF and atrial fibrillation.
• BNP, ANP, IL-6, TNF-α, and angiotensin II decreased with exercise.
• Aerobic training modulated inflammation and neurohormonal activity.
• No significant effect of exercise on arterial stiffness was observed.
• Randomized trial supports exercise in HFrEF with permanent AF.

## Introduction

Atrial Fibrillation (AF) is the most common sustained cardiac arrhythmia and substantially increases morbidity and mortality in patients with Heart Failure (HF) with reduced Ejection Fraction (HFrEF).^1^ The coexistence of HFrEF and AF is particularly detrimental because the two conditions interact synergistically to amplify systemic inflammation, neurohormonal activation, and arterial stiffness key mechanisms that contribute to hemodynamic deterioration and reduced functional capacity.^2^, ^3^, ^4^ Arterial stiffness, in particular, is exacerbated by endothelial dysfunction, oxidative stress, and chronic inflammatory signaling, all of which are heightened in patients with both HFrEF and AF.^3^^,^^4^ These vascular and inflammatory disturbances increase left ventricular afterload, impair coronary perfusion, and further aggravate diastolic dysfunction.^4^

Patients with HFrEF and AF often exhibit markedly reduced functional capacity due to combined impairments in cardiac output, peripheral vascular regulation, and oxygen utilization.^5^, ^6^, ^7^ Circulating inflammatory and neurohormonal biomarkers correlate inversely with exercise tolerance and may contribute to the pathophysiology of exercise intolerance in this population.^7^ Although exercise training improves functional capacity in HFrEF and AF separately, much less is known about whether exercise can modulate the heightened inflammatory and vascular burden unique to patients with both conditions.^5^, ^6^, ^7^, ^8^, ^9^ Exercise has the potential to reduce inflammation, improve endothelial function, and attenuate arterial stiffness; however, evidence specifically targeting the combined HFrEF and AF phenotype remains scarce and inconsistent.^3^^,^^4^^,^^8^^,^^9^

Given this knowledge gap, a better understanding of how exercise affects inflammatory and neurohormonal biomarkers and arterial stiffness in patients with HFrEF and permanent AF is needed. Therefore, this study investigated the effects of a 12-week aerobic exercise training program on biomarker levels and arterial stiffness in this high-risk population. Although the patient population overlaps with the parent randomized trial, none of the outcomes analyzed here were included in the primary publication.^5^ This secondary analysis focuses on mechanistic endpoints to provide complementary insights beyond the previously reported functional outcomes.

## Methods

### Study design and population

This investigation represents a pre-specified secondary analysis of the parent randomized clinical trial (NCT03550872). A detailed description of the study design, patient population, and interventions has been published previously.^5^ All patients had ischemic etiology, reflecting the characteristics of the population treated at our center during the enrollment period. The present analysis uses the same cohort but focuses exclusively on biomarker and arterial stiffness outcomes, which were predefined secondary endpoints. Importantly, none of the outcomes reported here were included in the primary publication, which centered on functional capacity. All procedures relevant to biomarker assessment and arterial stiffness measurement are detailed below to ensure methodological clarity for this secondary analysis.

**Fig. 1:**
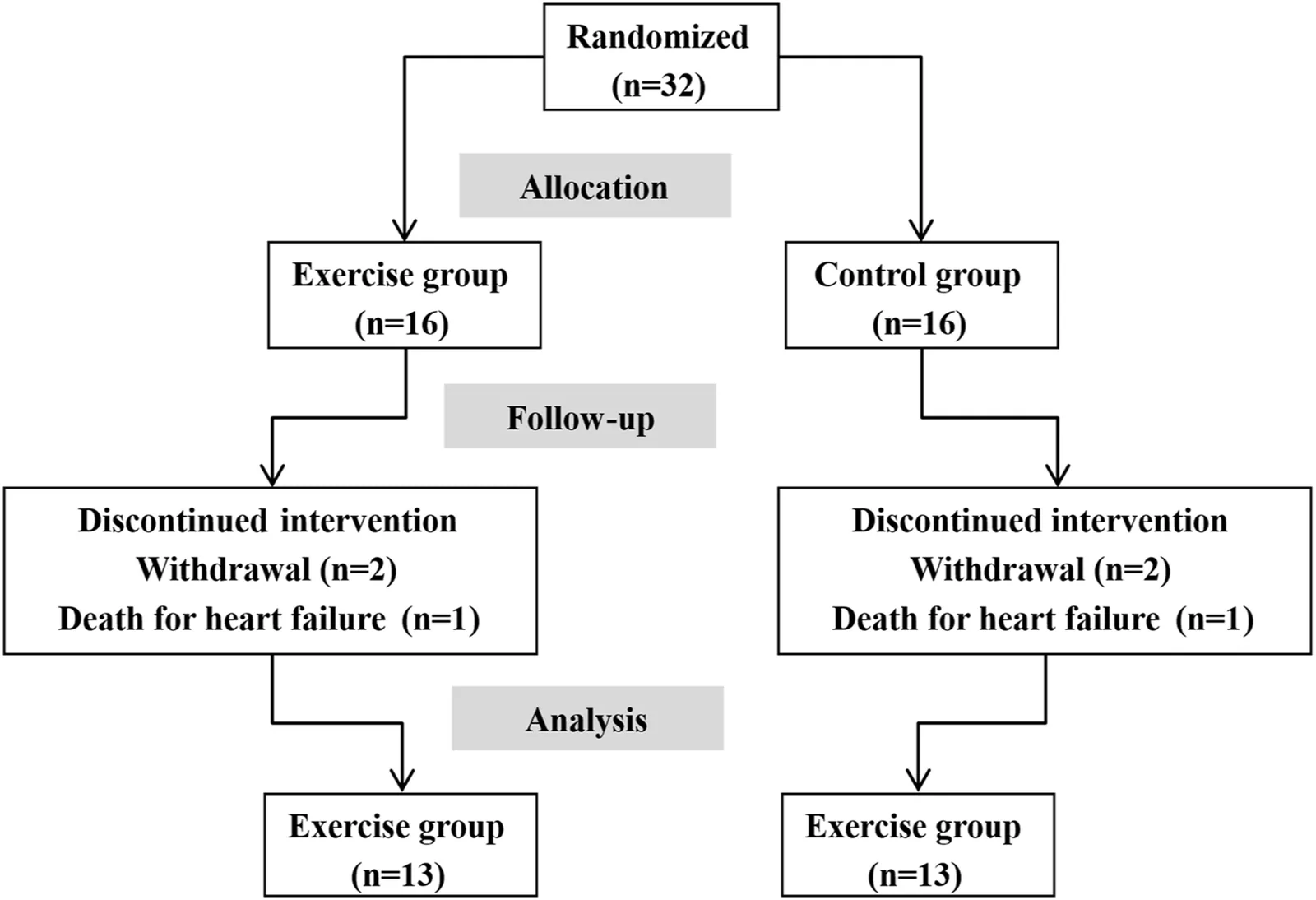
Comparison before and after 12-weeks of intervention between the exercise training group and control group. Arterial stiffness, p < 0.05, within-group comparison, and p = NS, Not Significant (NS) for between-group comparison (ANCOVA p = 0.15).

A brief description of the study design, patient population, and interventions is provided here: we recruited 32 patients with HFrEF and permanent atrial fibrillation, randomizing 1:1 to an exercise training group (n = 16) or a usual care control group (n = 16). Six participants discontinued the protocol (two dropouts and one death per group), resulting in a final analyzed cohort of 26 patients who completed the study (13 per group). These participants did not report any relevant adverse clinical signs or symptoms attributable to the intervention throughout the study (Fig. 1). The inclusion criteria at screening were age between 50- and 65-years, clinically stable, permanent atrial fibrillation (unsuccessful or indicated for rhythm reversal methods, such as ablation and cardioversion), resting heart rate ≤ 80 bpm, left ventricular ejection fraction ≤ 40 %, being under optimized clinical treatment and stable during the previous six weeks, and able to perform exercise training three times a week. The exclusion criteria were pulmonary disease, functional class IV (New York Heart Association [NYHA]), complex ventricular arrhythmia, chronic renal disease, peripheral neuropathy, history of stroke, Body Mass Index (BMI) > 30 kg/m^2^, history of smoking, and regular physical activity. Patients with a heart rate > 110 % of the age-predicted maximum heart rate during exercise testing and those implanted with a pacemaker, or an automatic implantable cardiac defibrillator were also excluded. The trial was approved by the Ethics Committee and was conducted in accordance with the Declaration of Helsinki. Written informed consent was obtained from all the patients.

This study was conducted and reported in accordance with the Consolidated Standards of Reporting Trials (CONSORT) 2010 Statement for randomized clinical trials. As the present manuscript represents a predefined secondary analysis of a randomized controlled trial, reporting was adapted to reflect the CONSORT recommendations applicable to secondary analyses of trial data.

### Outcomes

#### Arterial stiffness

Arterial stiffness was measured using carotid-femoral Pulse Wave Velocity (PWV) measurements with a noninvasive automatic device (SMT Medical GmbH & Co., Wuerzburg, Germany). The patients were asked to lie down in a quiet room and prone position after their BP and HR measurements were taken. The measurements were performed before (following a 10-minute seated rest), after, and during the recovery phase of each intervention by an experienced observer who was blinded to the intervention assignment, as previously described. Briefly, the pressure waveforms for the common carotid and femoral arteries were noninvasively estimated using a pressure-sensitive cuff. The distance between the recording sites (*d*) was measured using a flexible meter in a straight line, and PWV was automatically calculated as PWV = *d*/t, where is the pulse transit time.^10^

#### Biomarkers

Blood samples were obtained through venipuncture after a 20-minute rest period and an 8-hour fast. Samples were taken before the exercise program and between 18 and 72 h after the last session. The blood samples were immediately centrifuged at 2000 × g for 10-min at 4 °C, and the serum was stored at −70 °C until analysis.

B-type Natriuretic Peptide (BNP) was measured by immunoassay on a Beckman Access 2 Immunoassay Analyzer (Beckman Coulter, High Wycombe, UK) using Alere Triage BNP reagents (analytical range, 25–5000 pg/mL). Atrial Natriuretic Peptide (ANP) was measured by immunofluorescent assay on a Brahms Kryptor analyzer (Thermo Scientific, BRAHMS GmbH, Berlin, Germany; analytical range 2.1–10,000 pmol/L).

Interleukin-6 (IL-6), Tumor Necrosis Factor-alpha (TNF-alpha), and angiotensin II levels were measured using Multiplex MAP (Multi-Analytic Panels) kits and Luminex™ × MAP (Multiple Analyses Profiling) technology (EMD Millipore Corporation, Germany). The procedures were performed in accordance with the manufacturer’s instructions by an external laboratory that was blinded to the allocation group. All biomarkers were quantified using commercially available assay kits according to the manufacturers’ instructions. Assay precision was assessed by intra- and inter-assay coefficients of variation, which were approximately 1 % and 10 % for IL-6, 3 % and 19 % for TNF-α, and 5 % and 13 % for angiotensin II, respectively.

#### Exercise training

The exercise training program comprised three weekly sessions, each lasting 60-min, and was supervised for 12-weeks, as previously described.^5^ In brief, the exercise session consisted of 5-min of stretching exercises, followed by 40-min of cycling on an ergometer bicycle, 10-min of strengthening exercises, and finally, 5-min of cool down. The exercise intensity was prescribed using the Borg Scale and maintained between 14 and 16. Heart rate-based targets were not used because the irregular response characteristic of atrial fibrillation makes heart rate an unreliable parameter for prescribing intensity. Accordingly, perceived exertion was used as the primary criterion to guide exercise intensity.

### Statistical analysis

The original power calculation for the parent randomized clinical trial was based on detecting a 6 %–10 % increase in peak VO_2_. We estimated that a sample size of 13 participants per group would provide 85 % power to detect a 10 % change in peak VO_2_ with a two-sided α of 0.05. In the present manuscript, the biomarker and arterial stiffness outcomes represent predefined secondary analyses. Consequently, no independent a priori power calculation was conducted specifically for these secondary endpoints.

Data were analyzed using IBM SPSS Statistics (Version 17.0, IBM Corp., Armonk, NY, USA). Normality was assessed with the Shapiro Wilk test. Analyses were conducted using complete cases, with no imputation of missing values. Descriptive data are presented as mean ± Standard Deviation (SD). Baseline and post-intervention comparisons within groups were performed using paired-samples *t*-tests, and between-group pre-post differences (Δ) were assessed using independent-samples *t*-tests.

Between-group comparisons of post-intervention outcomes were performed using analysis of covariance (ANCOVA), with baseline values entered as covariates. This approach was used for all secondary outcomes and constituted the basis for all between-group inferences. Adjusted means, Standard Errors (SE), and 95 % Confidence Intervals (95 % CI) are reported.

Given the evaluation of six secondary outcomes, a Bonferroni correction was applied, yielding an adjusted significance threshold of α = 0.0083 (0.05/6). All p-values for secondary outcomes were interpreted relative to this corrected threshold. Under this criterion, the between-group difference in BNP remained statistically significant (p = 0.001), whereas the remaining outcomes did not meet the adjusted significance level and were therefore interpreted as non-significant.

## Results

A total of 26 patients completed the study, of which 13 were in the HFAF-trained group and 13 in the HFAF-untrained group. None of the participants reported any relevant clinical sign or symptom. The patients were clinically stable, and their medication was optimized, with the same medication maintained throughout the study at maximum tolerated doses as per the guidelines. No significant differences in baseline characteristics were found between the two groups.

**Fig. 2:**
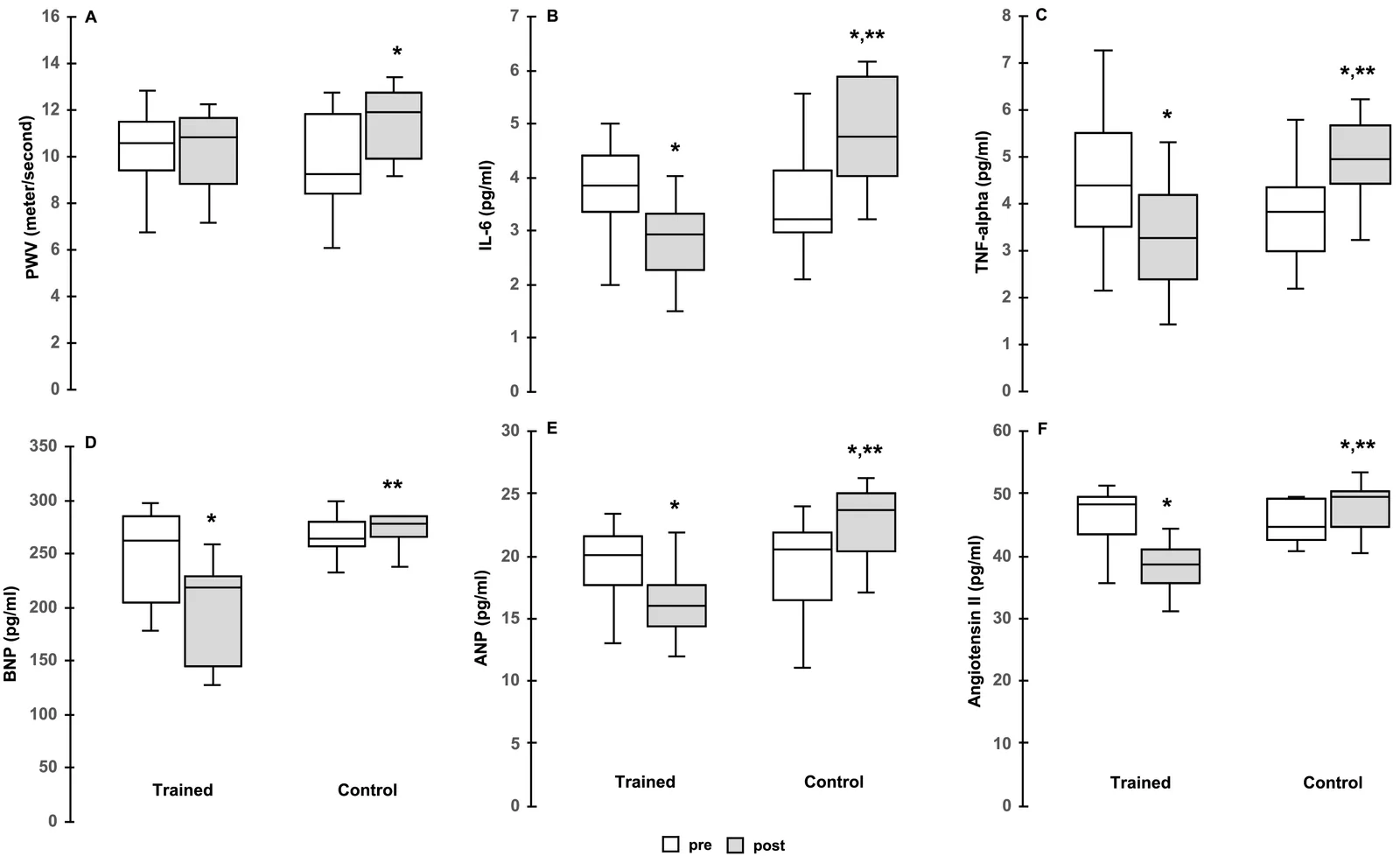
Comparison before and after 12-weeks of intervention between the exercise training group and control group. Biomarkers: (A) Interleukin-6; (B) Tumor Necrosis Factor-alpha; (C) B-type Natriuretic Peptide; (D) Atrial Natriuretic Peptide; and (E) Angiotensin II. *p < 0.05, within-group comparison, and **p < 0.0083, between-groups comparison.

At baseline, there were no differences between the groups in terms of arterial stiffness, BNP, ANP, IL-6, TNF-α, and angiotensin II (Table 1). We observed a significant worsening of arterial stiffness in the control group over time (from 9.7 ± 2.1 to 11.7 ± 2.2 m/s; p = 0.02) (Fig. 2A). In contrast, exercise training did not produce a significant change in arterial stiffness (from 10.7 ± 2.4 to 10.6 2.6 m/s; p = 0.86) (Fig. 2A). After adjustment for baseline values using ANCOVA, arterial stiffness did not differ significantly between groups (exercise: 10.4 ± 2.6 m/s; 95 % CI 9.0–11.8 vs. control: 11.9 ± 2.2 m/s; 95 % CI 10.5–13.3; *F* = 2.1, p = 0.15).

**Table 1:** Baseline characteristics according to the allocated group.

Parametrs	HFAF-UT	HFAF-T	p-value
	(n = 13)	(n = 13)	
Male sex	13	13	1.0
Age, years	58±3	58±2	0.5
BMI (kg/m^2^)	26±2.5	28±2.7	0.06
BSA (m^2^)	1.9 ± 0.1	2.0 ± 0.1	0.35
LVEF, %	32±5.5	34±4.7	0.73
LA, mm	54±4.7	53±7.6	0.26
Functional class, NYHA (%)			
II	4	6	0.42
III	9	7	
Etiology (%)			
Isquemic	13	13	1.0
Medications (%)			
ACEI ou ARB	11	13	0.14
Digoxin	10	8	0.40
Espironolactone	8	9	0.68
Amiodarone	1	1	1.0
Anlodipine	1	2	0.58
Beta-blocker	13	13	1.0
Diurétic	13	13	1.0
Estatíns	6	5	0.69
Anticoagulant	12	12	1.0
Hypoglycemic	5	6	0.69
Exercise			
Rest Systolic BP, mmHg	105±11.2	105 ± 14.1	0.34
Rest Diastolic BP, mmHg	64±13.1	65 ± 13.3	0.12
Rest HR, beats/min	72±6.3	75 ± 4.4	0.97
Peak HR, beatrs/min	148 ± 10.9	152 ± 8.2	0.41
Peak VO_2_ peak, mL.kg.min	14.2 ± 3.3	15.2 ± 2.2	0.43
VE/VCO_2_ slope	39 ± 6.3	39 ± 4.9	0.57

HFAF-UT, Patients with heart failure and atrial fibrillation untrained; HFAF-T, Patients with heart failure and atrial fibrillation trained; BMI, Body Mass Index; BSA, Body Surface Area; ACEI, Angiotensin Converting Enzyme Inhibitors; ARB, Angiotensin Receptor Blockers; BP, Bood Pressure; HR, Heart Rate; VO_2_, Oxygen uptake; VE/VCO_2_ slope, Relationship between ventilation and carbon dioxide output; LVEF, Left-Ventricular Ejection Fraction; LA, Left Atrium.

The exercise group exhibited a small, non-significant mean reduction pre-post in PWV (−0.14 m/s), whereas the control group showed a small, non-significant increase (+1.3 m/s). Although PWV increased significantly over time in the control group and remained unchanged in the exercise group, this between-group comparison yielded a null result, with no statistically significant difference in the magnitude of change between groups (p = 0.68). Collectively, these findings indicate that moderate-intensity exercise did not produce a statistically detectable effect on arterial stiffness over the study period.

Within the exercise group, significant pre–post reductions were observed in BNP (263.1 ± 74.4 to 195.1 ± 47.2 pg/mL; p = 0.01), ANP (46.3 ± 4.4 to 38.9 ± 4.6 pg/mL; p < 0.001), IL-6 (3.8 ± 0.83 to 2.8 ± 0.71 pg/mL; p < 0.001), TNF-α (4.5 ± 1.4 to 3.2 ± 1.1 pg/mL; p < 0.001), and angiotensin II (19.4 ± 3.1 to 16.3 ± 2.5 pg/mL; p < 0.001). In contrast, the control group exhibited significant increases in ANP (43.9 ± 3.1 to 46.4 ± 4.2 pg/mL; p = 0.004), IL-6 (4.0 ± 1.0 to 5.2 ± 1.8 pg/mL; p = 0.03), TNF-α (3.7 ± 1.1 to 5.0 ± 0.94 pg/mL; p = 0.002), and angiotensin II (19.9 ± 3.5 to 22.9 ± 3.2 pg/mL; p = 0.003) (Fig. 2 B-F).

After adjustment for baseline values using ANCOVA, significant between-group differences were observed for all circulating biomarkers, consistently favoring the exercise group. BNP levels were significantly lower in the exercise group compared with the control group (195.0 ± 47.2 pg/mL; 95 % CI 169.9–220.1 vs. 277.0 ± 26.9 pg/mL; 95 % CI 242.9–311.2; *F* = 16.6; p = 0.001). Similar between-group differences were observed for ANP (*F* = 66.5; p < 0.001), TNF-α (*F* = 65.2; p < 0.001), IL-6 (*F* = 33.0; p < 0.001), and angiotensin II (*F* = 66.5; p < 0.001). All differences remained statistically significant after Bonferroni correction (p < 0.0083).

Consistent with these findings, between-group differences in change scores demonstrated marked reductions favoring the exercise group for IL-6 (−0.9 vs. +1.1; p = 0.02), TNF-α (−1.1 vs. +1.2; p < 0.001), BNP (−68 vs. +9.1 pg/mL; p = 0.01), ANP (−7.3 vs. +2.5 pg/mL; p < 0.001).

## Discussion

This study examined the effects of a 12-week aerobic training program on inflammatory and neurohormonal biomarkers and arterial stiffness in patients with HFrEF and permanent atrial fibrillation a population characterized by complex hemodynamic, inflammatory, and vascular disturbances. Exercise training was associated with significant reductions in BNP, ANP, IL-6, TNF-α, and angiotensin II, whereas biomarker profiles deteriorated in the control group over the same period. When analyzed using baseline-adjusted between-group comparisons, these biomarker improvements remained statistically significant after correction for multiple comparisons, underscoring the biological relevance of exercise-induced modulation of pathways implicated in adverse cardiac remodeling in this high-risk phenotype.

In contrast, arterial stiffness demonstrated divergent within-group trajectories, with significant worsening in the control group and stability observed in the exercise group. However, the absence of a statistically significant between-group difference indicates that aerobic exercise did not produce a measurable effect on arterial stiffness relative to usual care over the study period. Accordingly, the present data do not support a protective or preventive effect of exercise on arterial stiffness in this cohort.

The biomarker improvements observed in the training group are consistent with prior work demonstrating that chronic aerobic exercise reduces systemic inflammation and dampens neurohormonal activation in cardiovascular disease.^7^^,^^11^^,^^12^ Patients with concomitant HFrEF and AF exhibit amplified cytokine production, elevated natriuretic peptide release, and heightened renin-angiotensin-aldosterone system activity; thus, the divergent biomarker trajectories in this study likely reflect meaningful modulation of disease activity.^7^^,^^13^^,^^14^ The progressive biomarker elevation in the control group underscores the ongoing pathophysiologic burden characteristic of HFrEF-AF.

In contrast, arterial stiffness did not improve with exercise training. Although pulse wave velocity increased in the control group and remained stable in the exercise group, the between-group difference was not statistically significant. Given the secondary nature of this endpoint and the limited statistical power of the study, these findings should be interpreted as a null result. Previous studies suggest that moderate-intensity exercise of limited duration may be insufficient to induce meaningful reductions in arterial stiffness in patients with advanced heart failure, with greater effects typically observed following higher training intensities, longer intervention periods, or greater exercise volumes.^15^, ^16^, ^17^, ^18^, ^19^, ^20^, ^21^ Accordingly, while the worsening observed in the control group is consistent with the progressive vascular dysfunction characteristic of HFrEF and atrial fibrillation,^22^, ^23^, ^24^, ^25^ the present data do not support a measurable effect of moderate-intensity exercise on arterial stiffness.

The results demonstrate that moderate-intensity exercise did not produce a detectable effect on arterial stiffness, with only minimal changes in either group. In contrast, exercise training led to clear and consistent improvements across multiple biomarker domains. IL-6 and TNF-α decreased substantially in the exercise group while increasing in controls, indicating a significant anti-inflammatory effect. Similarly, BNP and ANP showed marked reductions with training, whereas both increased in the control group, reflecting improved or stabilized cardiac stress physiology compared with progressive worsening in untrained patients. Angiotensin II followed the same pattern, decreasing with exercise and rising in controls, further supporting beneficial modulation of neurohormonal activation. Together, these findings suggest that while arterial stiffness remained unchanged, exercise training exerted meaningful anti-inflammatory and neurohormonal effects in patients with HFrEF and AF.

In conclusion, aerobic exercise training favorably modulated inflammatory and neurohormonal biomarkers in patients with HFrEF and permanent AF. However, no significant effect on arterial stiffness was observed, suggesting that moderate-intensity training may be insufficient to reverse established vascular stiffening. Future adequately powered trials incorporating higher-intensity or longer-duration exercise interventions are warranted to better elucidate vascular responsiveness in this complex clinical population.

## Clinical implications

Patients with HF and reduced ejection fraction who also have Atrial Fibrillation (AF) tend to experience a decline in their clinical status when their biomarker levels are elevated, even when receiving treatment according to current guidelines.^25^, ^26^, ^27^ However, this study demonstrated that exercise training can significantly lower biomarker levels in these patients. In contrast, untrained patients with HF and AF saw an increase in their biomarker levels.

Furthermore, arterial stiffness is linked to worsening hemodynamics, symptoms, and clinical outcomes in these individuals.^4^^,^^28^ While moderate-intensity exercise did not alter arterial stiffness, a worsening was observed in untrained patients. This study suggests that incorporating exercise training into the treatment strategy for these patients should be considered.

## Study limitations

Several limitations must be acknowledged. First, the sample size was modest and not originally powered for biomarker or arterial stiffness endpoints, reducing the ability to detect smaller effects. Second, this study represents a secondary analysis of a parent randomized trial, and therefore the findings should be interpreted within this context. Third, the inter-assay coefficients of variation for TNF-α and angiotensin II were relatively high, which may have introduced measurement variability. However, despite this variability, exercise training has been shown to reduce these specific biomarkers in individuals with heart failure.^7^ Finally, all participants had ischemic HFrEF and AF, the sample is relatively homogeneous, which may limit the generalizability of our findings to patients with non-ischemic etiologies.

## Conclusion

Aerobic exercise training favorably modulated inflammatory and neurohormonal biomarkers in patients with HFrEF and permanent AF. These biomarker findings complement our previous work demonstrating improvements in functional capacity and related physiological abnormalities in this population.^5^ No statistically significant between-group effect on arterial stiffness was observed, despite progression in the control group and stability in the exercise group. These exploratory findings from a secondary analysis suggest that exercise may help stabilize selected pathophysiological processes in this population, but they should be interpreted with caution. Larger, adequately powered trials are needed to confirm these results and clarify their clinical implications.

## Patient and public involvement

Patients and the public were not involved in the design, conduct, reporting, or dissemination of this research.

## Patient consent for publication

Not applicable.

## Authors’ contributions

GVG: Study concept and design; Analysis and interpretation of data, and manuscript preparation.

VMD: Study concept, design, and manuscript preparation.

PRC: Study concept, design, and manuscript preparation.

CA: Analysis and interpretation of data and manuscript preparation.

EAB: Study concept, design, and interpretation of data.

All authors gave their final approval and agreed to all aspects of the work, ensuring integrity and accuracy. The current study is presented honestly, without fabrication, falsification, or inappropriate data manipulation.

## Ethics approval

The study was approved by the Research Ethics Board of the Heart Institute and the Human Subject Protection Committee at the Clinics Hospital of the University of São Paulo Medical School (SDC 3972/13/097), adhered to the Declaration of Helsinki, and registered on ClinicalTrials.gov (NCT03550872). All the participants provided written informed consent.

## Funding

This work was supported by the Fundação de Amparo à Pesquisa do Estado de São Paulo (FAPESP # 2013/17031-6).

## Declaration of competing interests

The author(s) declare no potential conflicts of interest regarding this article's research, authorship, or publication.

## Data Availability

Data from this article will be shared upon reasonable request with the corresponding author.
